# Anything You Can Do, You Can Do Better: Neural Substrates of Incentive-Based Performance Enhancement

**DOI:** 10.1371/journal.pbio.1001272

**Published:** 2012-02-21

**Authors:** Mimi Liljeholm, John P. O'Doherty

**Affiliations:** Division of the Humanities and Social Sciences and Computation and Neural Systems Program, California Institute of Technology, Pasadena, California, United States of America

## Abstract

Performance-based pay schemes in many organizations share the fundamental assumption that the performance level for a given task will increase as a function of the amount of incentive provided. Consistent with this notion, psychological studies have demonstrated that expectations of reward can improve performance on a plethora of different cognitive and physical tasks, ranging from problem solving to the voluntary regulation of heart rate. However, much less is understood about the neural mechanisms of incentivized performance enhancement. In particular, it is still an open question how brain areas that encode expectations about reward are able to translate incentives into improved performance across fundamentally different cognitive and physical task requirements.

## Abstract 2

A neuroimaging study reveals that both mental and physical effort might be motivated by a single neural module located in deep sub-cortical structures.

## Incentives Modulate Performance across Task Domains

Previous work indicates that reward anticipation in the human brain is mediated by an interconnecting network of cortical and sub-cortical structures, incorporating the ventromedial prefrontal cortex, the amygdala, and the ventral striatum (VS) [1]. Furthermore, activity in at least one of these structures, the VS, appears to be specifically related to performance enhancement in response to incentives across a range of psychological tasks. For instance, Pessiglione et al. [2] found that the amount of physical force exerted on a hand- grip increased with the amount of monetary reward contingent on reaching a criterion force level. They also found that while activity in motor regions increased with the amount of force, activity in the VS increased with the magnitude of the anticipated reward. In addition, they showed that the VS was responsible for the modulation of motor behavior. Further evidence implicating the VS in translating incentives into enhanced performance comes from a study by Pleger et al. [3], in which it was found that the accuracy of tactile discriminations increased with the amount of monetary reward that followed correct performance. At the neural level, activity in the VS again increased with increasing incentives, as did activity in the task-relevant somatosensory primary cortex.

Interestingly, studies that assess the influence of incentives on high level cognition have reported effects that are strikingly similar to those emerging in sensory and motor tasks. In a recent study by Krebs et al. [4], participants were scanned as they performed a Stroop task, in which they had to press specific buttons to indicate the ink color of words presented on the screen. As is typical with this task, incongruent words (spelling out a different color than the words' actual color) impaired performance relative to congruent words. However, in Krebs et al.'s study, some of the ink colors were associated with monetary reward or penalty, contingent on the speed and accuracy of performance on a given trial. Fewer errors and faster response times were observed for ink colors associated with potential reward, in both congruent and incongruent conditions, suggesting that the presence of a monetary incentive produced a general improvement in performance. Consistent with the results described above, Krebs et al. found that activity in the VS was greater for ink colors associated with potential reward. These reward-related increases in VS activity were correlated with the reward-induced facilitation of performance.

## A Common Motivational Node for Cognitive and Sensorimotor Systems

**Figure 1:**
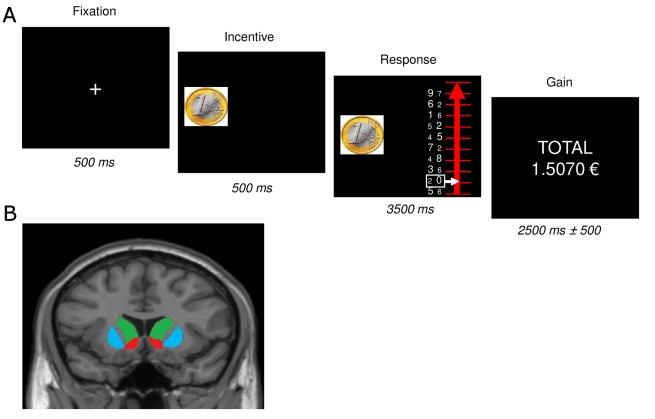
Illustration of Schmidt et al.'s task, and of the striatum. (A) Trial structure in Schmidt et al.'s study. On each trial, after a brief fixation period, participants were shown the monetary incentive on that trial (either high or low). The subsequent screen showed a graduated line, with each graduation corresponding to obtainment of 10% of the previously shown coin, and with a pair of digits of different numerical and physical sizes printed next to each graduation. (B) Coronal slice through the human brain, showing the location of the VS, caudate, and putamen in red, green, and blue, respectively.

Evidence from neuroimaging studies assessing the role of incentives in cognitive control on the one hand [4], and sensory and motor performance on the other [2],[3], suggests the VS is a common motivational node that flexibly interacts with distinct cortical networks, thus translating incentives into enhanced performance across domains. However, at the level of separate studies, it cannot be determined whether the encoding of reward expectation in the VS, and the corollary effects on performance, are truly equivalent for both cognitive and motor tasks. In a new study published in *PLoS Biology*, Schmidt et al. [5] directly tackle this issue of task specificity by contrasting cognitive and physical incentivized efforts in a single experiment, and even on a single trial. In their task, on each trial, individuals were presented with a graduated line, where each graduation represented obtainment of 10% of a previously shown monetary reward, and corresponded to a pair of digits, one always numerically smaller than the other (see Figure 1A).

Participants had to squeeze one of two hand-grips (left or right) in order to move a cursor as far up the ladder as possible; the further the cursor went, the larger the reward. The required grip for each graduation was indicated by the left/right location of the numerically smaller of the two digits printed next to the graduation; for incongruent digit pairs, the number printed in the greater font size was that which was numerically smaller; conversely, for congruent pairs, a single digit was smaller both numerically and in terms of size. Schmidt et al. independently varied cognitive effort (the frequency of incongruent number pairs) and physical effort (the force required to move the cursor one graduation using the relevant hand-grip), as well as the monetary incentive, which was indicated by a coin image displayed at the onset of each trial.

Consistent with the notion of the VS as a common motivational node, Schmidt et al. found that activity in this area increased with expected reward, and correlated with performance across cognitive and physical effort requirements. Critically, using dynamic causal modeling—a technique aimed at determining how interactions between brain areas change across experimental contexts—they also found that the VS selectively enhanced activity in motor and cognitive sub-cortical regions, the putamen and caudate, respectively (see Figure 1B), according to task demand. While it is still possible that dedicated sub-regions in the VS, falling below the threshold of spatial resolution afforded by functional MRI, encode incentives separately for cognitive and physical effort, the carefully controlled comparison carried out by Schmidt et al. strongly suggests that, at the macroscopic level, this structure encodes a single, multipurpose, value signal that is used for the specific task at hand.

## Outstanding Questions

### The Role of Associative Mechanisms

So far, we have considered only the incentive effects of stimuli or conditions that indicate that reward is contingent on performance in a task. However, a substantial body of research has focused on the influence of cues signaling rewards that are independent of any actions performed by the subject, a phenomenon referred to as Pavlovian-instrumental transfer (PIT). In a typical PIT experiment, subjects are trained on a Pavlovian relationship (i.e., stimulus→reward) as well as, separately, on an instrumental relationship (i.e., action→reward); in a subsequent test, instrumental responding is greater in the presence of the reward-paired stimulus than in the presence of a control stimulus that had also been previously presented but not paired with reward. The increase in instrumental responding occurs even if the reward-paired stimulus predicts a different reward than that predicted by the instrumental action, and is attenuated by shifts from hunger to satiety. These results are consistent with the idea that reward predicting cues induce a general motivational state that, in turn, invigorates instrumental performance.

The PIT effect has been demonstrated in both rodents [6],[7] and humans [8],[9], and has been shown to involve the VS in both species (see [10] for a review). An important question is whether the results obtained by Schmidt et al. might be, wholly or partly, due to PIT, in which case they should also have been observed if the reward stimulus (i.e., the coin displayed at trial onset) had been unrelated to the magnitude of performance-contingent reward. There are at least two other reward-related variables worth considering as sources of the VS activity observed by Schmidt et al. Contemporary theories of behavioral control distinguish between *goal-directed* actions, which are selected based on deliberate consideration of their consequences, and *habits*, which are more reflexively elicited by their stimulus environment [11]–[13]. In Schmidt et al.'s study, the signal encoded by the VS may have reflected either a goal-directed consideration of the consequences of performing an action, or the strength of habitual action–elicitation by high- and low-incentive cues. Further work is needed to determine how various associative processes relate to the role of the VS in performance modulation.

### Delineating the Neuronal Responses within the Ventral Striatum

The finding that the VS is acting as a common motivational node mediating effects of incentive on both motor and cognitive performance leaves many open questions as to the precise neural mechanisms by which this process occurs within this structure. A considerable body of work has focused on the possible contributing role of dopamine neurons, which densely innervate the VS [14]. Dopamine release in the VS in response to incentive cues has been shown to vary more closely with the magnitude of anticipated reward than with costs, such as those associated with effort, although some evidence for modulation by cost has been reported [15],[16]. However, a fuller understanding of how the VS mediates performance modulation will ultimately require more fine-grained neurophysiological measurement of intrinsic neuronal activity within the VS itself, as well as a much more detailed characterization of the nature of the interactions between VS neurons and neurons involved in implementing cognitive and motor behavior. The promising new findings by Schmidt et al. [5] provide fresh motivation for our efforts to unravel the computational and neural processes underpinning the modulation of performance by incentives.

## Abbreviations

PIT: Pavlovian-instrumental transfer
VS: ventral striatum
